# Controlling Magnetic-Field-Induced Shape Memory Response in Polycrystalline Off-Stoichiometry Fe_47‑x_Mn_24**+**x_Ga_29_ Microwires

**DOI:** 10.1021/acsmaterialsau.5c00113

**Published:** 2025-08-29

**Authors:** Reddithota Vidyasagar, Michal Varga, Pavel Diko, Tomas Ryba, Pablo Rafael Trajano Ribeiro, Fernando Luis de Araujo Machado, Snorri Thorgeir Ingvarsson, Rastislav Varga

**Affiliations:** † Centre of Progressive Materials, TIP, Pavol Jozef Safarik University in Kosice, Tr. SNP 1, Kosice 040 01, Slovak Republic; ‡ Science institute, University of Iceland, Dunhaga 3, Reykjavik IS-107, Iceland; § Faculty of Materials, 87276 Metallurgy and Recycling at the Technical University of Kosice, Letna 9, Kosice 042 00, Slovak Republic; ∥ Institute of Experimental Physics, 193011 Slovak Academy of Sciences, Watsonova 47, Košice 040 01, Slovak Republic; ⊥ RVmagnetics, Nemcovej 30, Kosice 04001, Slovakia; # Departamento de Física, 133639 Universidade Federal de Pernambuco, Recife, Pernambuco 50670-901 Brazil; ○ Institute of Physics, Czech Academy of Sciences, Na Slovance 2, 182 00 Praha 8, Czech Republic

**Keywords:** Fe-based Heusler alloys, magnetic shape memory, Martensitic transformation, L2_1_ structure, ac magnetic susceptibility, magnetic hysteresis

## Abstract

The ferromagnetic shape memory (FSM) behavior of glass-coated Fe_47‑*x*_Mn_24+*x*_Ga_29_ (x = 0–8 at. %) microwires has been investigated through temperature-dependent magnetization and *ac* magnetic susceptibility measurements. Magnetization measurements as a function of temperature reveal an abrupt increase and decrease in magnetization upon cooling and heating, respectively, indicating characteristic thermal hysteresis (*ΔT*_*hys*_) behavior typically associated with a magnetic-field-induced “diffusionless” martensitic transformation. The magnitude and width of *ΔT*_*hys*_ are strongly correlated with the Fe/Mn atomic ratio; notably, the Fe_45_Mn_26_Ga_29_ microwire exhibits a very large *ΔT*_*hys*_ width of 98 K, which is attributed to local deformation involving the motion of Fe and Mn atoms. Furthermore, an antiferromagnetic transition is observed in a low-temperature region, shifting from 22 to 41 K depending on composition. This shift is attributed to variations in local exchange interactions arising from unequal occupation of Fe and Mn 3*d* orbitals. These findings highlight a compositionally driven design strategy that enables precise tuning of FSM behavior, making Fe–Mn–Ga microwires promising candidates for use in tunable magnetic actuation and sensing technologies.

## Graphical abstract

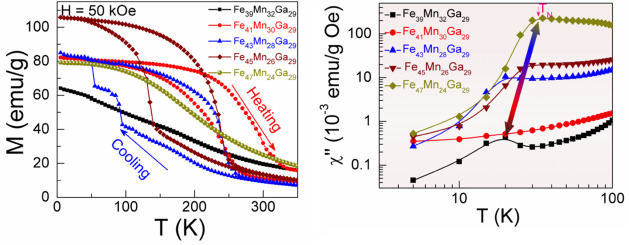

## 1 Introduction

The class of Heusler alloys is well known for exhibiting a strong structure–property relation, attracting significant attention not only from the solid-state physics viewpoint but also because of their practical device applications.^1–4^ The interplay between crystal structure and electronic spin ordering within a single material is expected to give rise to novel phenomena such as magneto-structural,^5–7^ magneto-electric,^8^ electro-structural,^8,9^ and other competing coupled effects. These arise from the interaction between the magnetic moment and electric resistivity, particularly during structural symmetry transformations. Nevertheless, only a few synthetic alloys have been found to exhibit both magneto-structural and magneto-electric properties. Although several approaches are currently being pursued for designing bulk Fe-based Heusler alloys, the Taylor–Ulitovski technique has been employed effectively and did not only lay out the ∼100 μm wire covered with a protective glass layer but also easily integrated to engineering and medical devices.^10–12^ Furthermore, this method ensures a near-stoichiometric distribution of elemental components, making it compatible with the composition of the starting materials. Indeed, prior theoretical and experimental studies have demonstrated magnetic shape memory response in Fe–Mn–Ga alloys, indicating that their physical mechanisms driving the magnetic-field-induced martensitic phase transformation are extremely sensitive to temperature, local structural environment of magnetic atoms, number of valence electrons, and compositional design. These findings suggest that Fe-based Heusler compounds hold great promise for the development of next-generation FSM devices.

Despite the stabilization of the complete phase sequence of martensitic ↔ austenite phase transition in off-stoichiometric Fe-based Heusler compounds, Fe–Mn–Ga alloys allow control through external means such as magnetic fields and temperature, as well as internal means like composition. The control of FSM behavior in Fe-based Heusler alloys can be categorized into two distinct compositional design strategies: (i) chemical doping is often employed as a nonthermal control parameter to tune Fe-based Heusler materials across the magnetic-field-induced martensitic transformation. While this approach has been proven effective in some cases, it presents several challenges. The incorporation of foreign atoms into the Heusler structure is inherently a discrete rather than continuous tuning variable. Moreover, doping frequently introduces competing quenched structural disorder, which can disrupt the delicate balance between the structural and magnetic order. As a result, the shape memory response of the material may be significantly altered in poorly controlled ways.^2–4^ (ii) In the Fe–Mn–Ga Heusler alloys, the FSM effect is often tuned by substituting Ga with Fe, Mn, or other transition metals.^13–15^ This doping strategy is considered effective because it introduces lattice distortions arising from the atomic size mismatch, specifically, between Fe (0.140 nm) and Ga (0.130 nm), which can potentially influence the FMS response. However, this approach adds complexity, as both atomic site occupancy (which induces local lattice distortion) and exchange interactions—especially between Mn and Ga—play critical roles in determining the FSM behavior.^5–8^ As a result, the underlying physical mechanism governing the FSM control remains ambiguous. While doping introduces both structural and magnetic modifications, a clear separation of the effects of lattice distortion and magnetic exchange interactions is still lacking. Interestingly, because Fe and Mn have nearly identical atomic radii, it is possible to control the FSM response through localized magnetic interactions without significantly altering the crystal structure.^16^ This allows for a more isolated study of magnetic contributions to the FSM effect. Nonetheless, the kinetic mechanism underlying the FSM behavior, particularly the emergence of a low-temperature antiferromagnetic state in relation to varying Fe–Mn ratios in Fe–Mn–Ga alloys, remains poorly understood, in contrast to Ni-based Heusler alloys that have been studied in detail.^14–18^ These observations underscore the urgent need for a systematic and detailed investigation into compositionally controlled, reproducible Fe–Mn–Ga microwires in order to fully understand and exploit their functional potential. In this study, we report the synthesis of high-quality Fe–Mn–Ga Heusler microwires with systematically varied Fe and Mn volume proportions to investigate their influence on the FMS behavior. X-ray diffraction measurements shows that the polycrystalline Fe_47‑x_Mn_24+x_Ga_29_ Heusler alloy’s lattice parameter very moderately changes (less than 1%) with *x* = 8, 6, 4, 2, and 0 at. %. Apart from the negligible lattice distortion in Fe_47‑x_Mn_24+x_Ga_29_ microwires, the magnetization behavior during cooling and heating cycles exhibits thermal hysteresis, indicative of a magnetic-field-induced martensitic transformation. The magnitude and shape of this thermal hysteresis are significantly influenced by competing Fe–Mn interactions, particularly for the compositions with *x* = 6, 4, and 2 at. %. Furthermore, *ac* susceptibility measurements reveal the presence of a low-temperature antiferromagnetic (AFM) transition. This transition is attributed to the competing exchange interactions—specifically, ferromagnetic coupling among equivalent Mn atoms and AFM interaction between Mn and Fe atoms—modulated by the arrangement of Mn–Fe planes within the Fe–Mn–Ga lattice.

## 2 Glass-Coated Microwires and Measurements

Button-type ingots (10 g each) of polycrystalline Fe_47‑x_Mn_24+x_Ga_29_ Heusler alloys (*x* = 8, 6, 4, 2, and 0 at. %) were prepared via *arc* melting stoichiometric amounts of 5 N purity elemental of *Fe granules, Mn pieces*, and *Ga pieces*. The melting was carried out under a high-purity argon atmosphere on a water-cooled copper electrode using an Edmund Buhler MAM-1 mini-arc melting system. To ensure chemical homogeneity, each alloy was remelted five times under an argon environment maintained at a vacuum of 10^–2^ Torr. Subsequently, the ingots were processed into glass-coated microwires using the Taylor–Ulitovsky technique, which involves induction melting and drawing of the alloys into a Pyrex glass-coated wire. This method yields a continuous, stoichiometric metallic core coated in glass, preserving the composition of the starting materials.

The surface morphology and homogeneous chemical composition of the metallic core of the wires were analyzed at room temperature using a scanning electron microscope (SEM, model: MIRA3 TESCAN) equipped with an energy-dispersive X-ray spectroscopy (EDS) system (*x*-act EDS microanalyzer, Oxford Instruments). X-ray diffraction (XRD) patterns of Fe_47‑x_Mn_24+x_Ga_29_ alloys (with *x* = 8, 6, 4, 2, and 0 at. %) were recorded using a Rigaku D/MAX RAPID diffractometer, with MoKα radiation and two-dimensional curved detector. The sample-to-detector distance was approximately 124.7 mm, and the X-ray beam width was 100 μm, defined by a collimator. The measurements were conducted in transmission geometry on as-cast alloy samples with sufficient thickness for X-ray transmission. Magnetic measurements, including *dc* magnetization (*M*) and *ac* susceptibility were performed using the *ACMS* modulus of the Physical Properties Measurements System by Quantum Design (PPMS). Temperature-dependent magnetization was recorded during both cooling and heating processes under various applied magnetic fields (*H*) of 0.05, 5, 10, 20, and 50 kOe for all alloy compositions. The *ac* susceptibility measurements were carried out in both in-plane and out-of-plane configurations using an *ac* magnetic field with a frequency of 1 kHz and an amplitude of 10 Oe, in the temperature range of 5–350 K. Magnetic hysteresis loops were measured by sweeping the magnetic field (±85 kOe) at selected temperatures of *T* = 5, 150, and 300 K. These experiments provide direct evidence of the magnetically induced FMS response in off-stoichiometry Fe-based Heusler alloys, which can be tuned via chemical composition.

## 3 Results and Discussion

Figure 1a-e presents SEM images of as-prepared glass-coated Fe_47‑x_Mn_24+x_Ga_29_ (*x* = 8, 6, 4, 2, and 0 at. %) microwires prepared via the Taylor–Ulitovski technique. These images clearly reveal that the components of nonstoichiometric glass-coated Fe_47x_Mn_24+x_Ga_29_ microwires are uniformly distributed, exhibiting no visible damage or cracks. The metallic core is spatially confined within protective glass coating, which significantly reduces interaction with the external environment and thus enhances the physical stability of the samples without chemical bond breakings. The total diameter of the glass-coated microwires ranges from approximately *D*≈125 to 250 μm, while the metallic core diameter falls within *d*≈50–100 μm. Energy-dispersive X-ray spectroscopy (EDS) analysis, conducted over a large area of individual microwires, confirms the relatively uniform elemental distribution with no evident surface segregation of the alloying constituents. The presence and relative proportions of Fe, Mn, and Ga are confirmed by the EDS profiles, and the quantified elemental compositions are summarized in Table 1. The surface enrichment of Fe (as indicated in Table 1) and the corresponding depletion of Mn atomic percentage occur, while Ga remains almost constant at approximately 29 ± 1 at. %. This surface segregation of Fe and Mn could act as pinning centers for domain walls, thereby inhibiting their motion. Taking into account the intrinsic quantification limitations of EDS, the measured compositions are in reasonable agreement with the nominal values of precursor mater alloys. It can be concluded that the compositional elemental values reasonably agree well with the nominal composition values of the master alloys.

**Figure 1:**
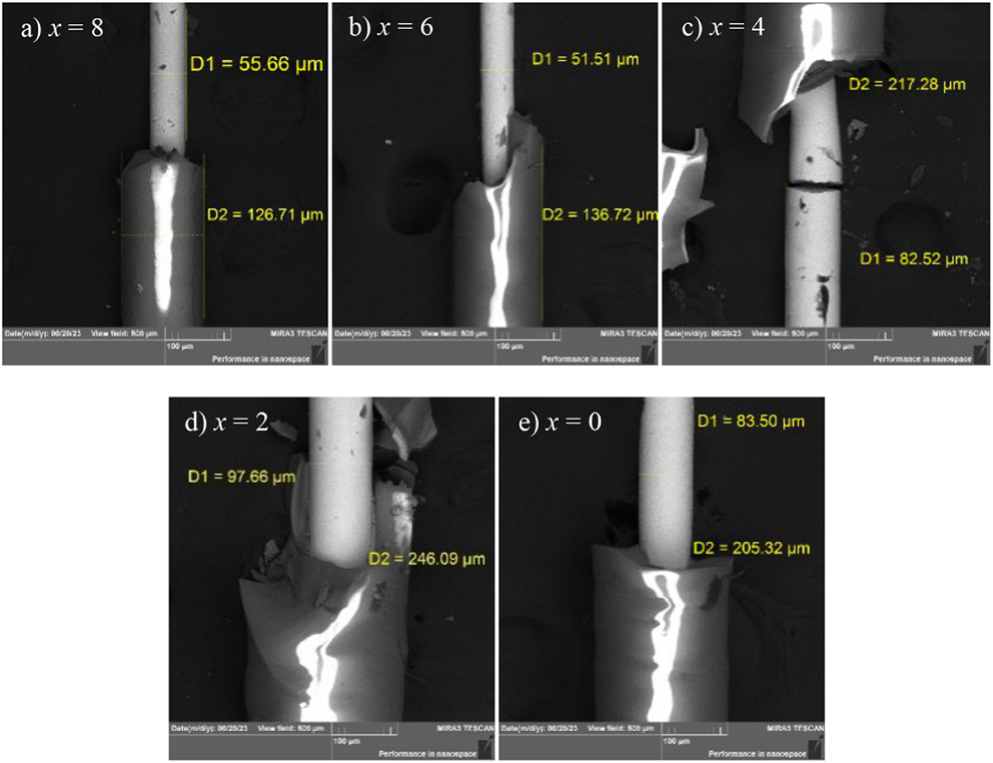
(a-e) Scanning electron microscope images of glass-coated Fe_47‑x_Mn_24+x_Ga_29_ microwires prepared by the Taylor–Ulitovsky method. The glass-coated microwire diameter and metallic nucleus diameter values are shown inside the images.

**Table 1:** EDS Composition of Glass-Coated Fe_47‑x_Mn_24+x_Ga_29_ Microwires

		Nominal composition (at. %)			Composition according to EDS experiments (at. %)		
S. No	Heusler alloy microwire	Fe	Mn	Ga	Fe	Mn	Ga
**1**	Fe_39_Mn_32_Ga_29_	39	32	29	39.9	31.6	28.5
**2**	Fe_41_Mn_30_Ga_29_	41	30	29	40.8	30.9	28.3
**3**	Fe_43_Mn_28_Ga_29_	43	28	29	43.0	26.3	30.7
**4**	Fe_45_Mn_26_Ga_29_	45	26	29	44.9	26.3	28.8
**5**	Fe_47_Mn_24_Ga_29_	47	24	29	47.4	23.2	29.4

Figure 2a shows the stacked X-ray diffraction (XRD) patterns of as-synthesized glass-coated microsized Fe_47x_Mn_24+x_Ga_29_ microwires with compositions *x* = 8, 6, 4, 2, and 0 at. %. All microwires exhibited main diffraction peaks of (220), (400), (422), (440), and (620) planes. These reflections follow the selection rules of space group No. 225, *Fm3̅m*, in which only reflections with all-even or all-odd Miller indices (*hkl*) are allowed. This confirms that the microwires possess a pure single-phase *face-centered cubic (fcc)* L2_1_ structure. The experimental lattice constants were found to be 5.879, 5.876, 5.862, 5.855, and 5.822 Å, respectively, as shown in Figure 2b. These values correspond to a gradual shift in diffraction angle from 2θ = 19.69̊ to 19.85̊, consistent with the expected behavior of the L2_1_ unit cell. The lattice constants are in good agreement with both theoretical and experimental data previously reported in the literature.^5,19,20^ As the Mn concentration decreases (from 8 to 0 at. %) by substitution with Fe, the lattice parameter moderately decreases from 5.879 to 5.822 Å. Although the atomic radii of Mn (0.127 nm) and Fe (0.126 nm) are nearly identical, this reduction in the lattice parameter indicates minor lattice contraction. This is likely due to Fe–Mn interactions and the slight nonstoichiometry in Ga (∼1 atom %), rather than size effects alone. Thus, the Mn atoms are understood to substitute for Fe without altering the *fcc* L2_1_ crystal structure. The resulting unit cell volume decreases by approximately 2.9%, which may also involve slight atomic displacements. Previous XRD studies on Fe–Mn–Ga alloys indicate the coexistence of a high-temperature *fcc* L2_1_ austenite phase (*a* = 5.88 Å) and low-temperature *bcc* L1_2_ martensite phase (*a* = 3.71 Å).^11,19,20^ Although the high-temperature austenite phase is known to have a cubic L2_1_ structure (space group No. 225, Fm3̅m), the crystal structure of the low-temperature ferromagnetic martensitic phase remains controversial. Previous studies on Fe_43_Mn_28_Ga_29_ microwire composition confirmed the coexistence of L2_1_ and L1_2_ structures.^11,20^ Based on this, we initially presumed that the low-temperature martensitic phase corresponds to the L1_2_ structure. Nevertheless, prior XRD experiments have shown that the low-temperature ferromagnetic phase exhibits a tetragonally distorted structure for the low-temperature ferromagnetic martensitic phase.^5^ In particular, previous temperature-dependent XRD measurements on bulk Fe_44‑*x*_Mn_28_Ga_28+*x*_ ingots have shown that the samples undergo a structural transformation from the cubic austenitic L2_1_ phase to a tetragonal martensitic phase, which has been reported either as the body-centered tetragonal structure (space group No. 139, *I*4/*mmm*)^14^ or as the face-centered tetragonal L1_0_ structure.^5^ We agree with the reviewer that the presence of the L1_2_ structure at low temperatures is not conclusively evidenced. According to prior experimental reports, the martensitic phase is more accurately described as a tetragonally distorted structure. The martensitic transformation would induce a large compressive strain of ∼30% relative to the austenite phase. Such strain is associated with strong orbital overlap between Fe-3*d* and Mn-3*d* electrons, which can enhance exchange interactions when a magnetic field is applied.

**Figure 2:**
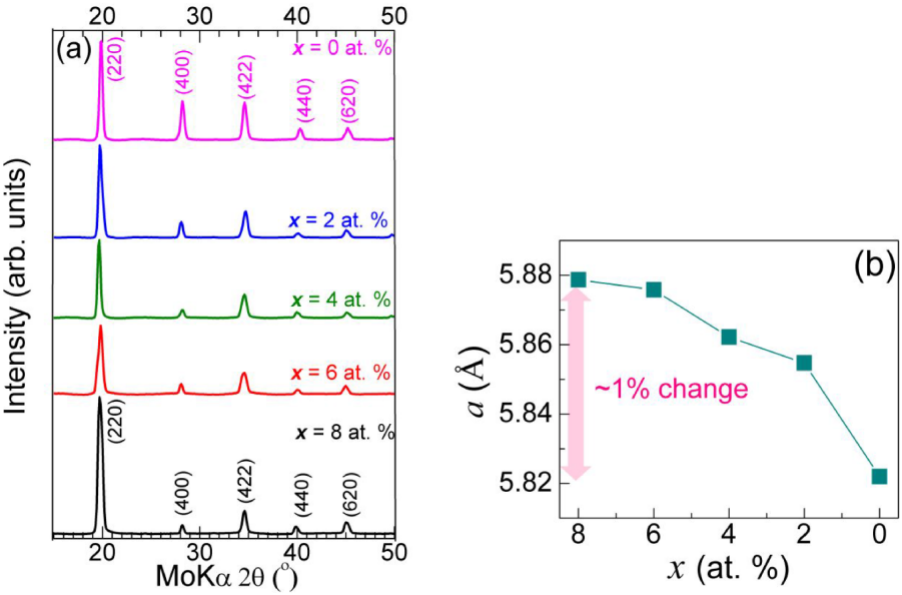
(a) Stacked XRD patterns of the glass-coated Fe_47x_Mn_24+x_Ga_29_ (*x* = 8, 6, 4, 2, and 0 at. %) microwires indicating the shift of the most prominent (220) peak. (b) The lattice parameter of Fe_47x_Mn_24+x_Ga_29_ as a function of Mn content suggests that the lattice is reduced about ∼1% with decreases of Mn content.

### 3.1 Thermal Hysteresis Characteristics

To investigate the thermal and magnetic field memory responses associated with the martensite-to-austenite phase transformation, we performed temperature- and field-dependent magnetization measurements of off-stoichiometric Heusler-type Fe_47‑x_Mn_24+x_Ga_29_ microwires. Figure 3a–e presents the temperature dependence of magnetization, *M­(T)*, for glass-coated Fe_47‑x_Mn_24+x_Ga_29_ microwires under applied magnetic fields of *H* = 0.05, 5, 10, 20, and 50 kOe, oriented along the longitudinal axis of microwire. The *M­(T)* curves measured during both cooling and heating cycles for *x* = 8 and 0 at. % follow the same path, indicating the absence of thermal hysteresis (*ΔT*_*hys*_), even under magnetic fields up to 50 kOe. This behavior suggests that a magneto-structural transformation, typically associated with the low-temperature ferromagnetic martensitic phase, does not occur in these compositions. The spontaneous magnetization observed below 180 K originates from unbalanced antiparallel spin configurations, resulting in a net magnetic moment that is not associated with structural phase transformations.

**Figure 3:**
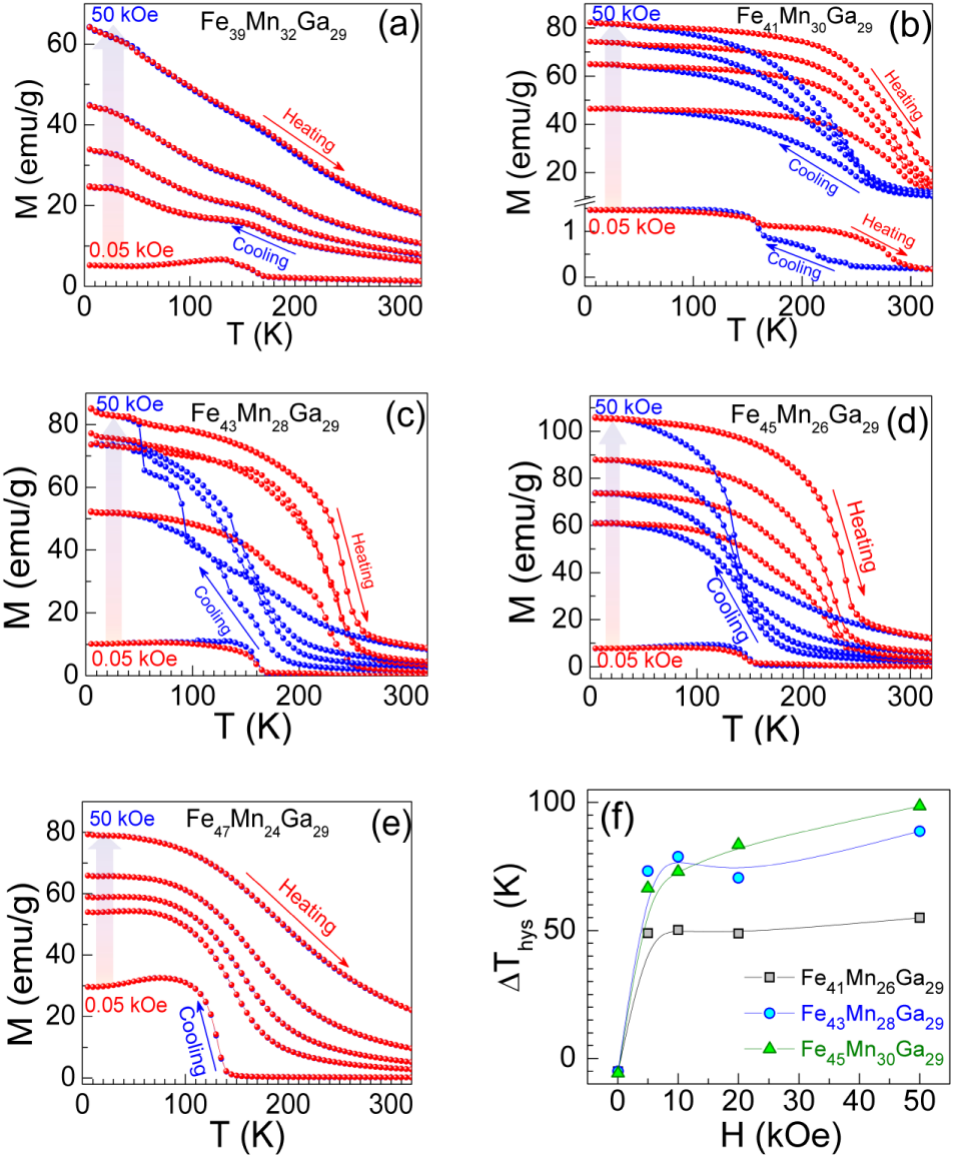
(a-e) Thermo-magnetization curves of crystalline Fe_47‑*x*_Mn_24+*x*_Ga_29_ (*x* = 8, 6, 4, 2, and 0 at. %) microwires measured in the applied magnetic field of H= 0.05, 5, 10, 20, and 50 kOe along the length of microwire. (f) Thermal hysteresis *ΔT*_*hys*_ (K) width as a function of magnetic field (*H*) for Fe_41_Mn_30_Ga_29_, Fe_43_Mn_28_Ga_29_, and Fe_45_Mn_26_Ga_29_ for microwires.

Figure 3b–d shows the *M­(T)* curves for microwires with compositions *x* = 6, 4, and 2 at. %, measured under magnetic fields of *H* = 0.05, 5, 10, 20, and 50 kOe. Remarkably, the microwires exhibit an abrupt increase and a subsequent decrease of spontaneous magnetization during cooling and heating cycles, respectively. This behavior clearly indicates the presence of thermal hysteresis (*ΔT*_*hys*_), which is a hallmark of the first-order, diffusionless martensitic transformation.^5,8^ According to previous studies, such behavior corresponds to coupled magneto-structural phase transitions between a low-temperature ferromagnetic martensite phase and a high-temperature paramagnetic austenite phase.^21^ This thermal hysteresis is typically defined as *ΔT*_*hys*_ = T_Af_ - T_Ms_ (or alternatively T_As_ - T_Mf_), where T_Ms_ and T_Mf_ are the start and finish temperatures of the martensitic transformation, respectively, and T_As_ and T_Af_ are the corresponding temperatures for the reverse austenitic transformation.^22^ From Figure 3b, it is evident that both the magnitude and shape of thermal hysteresis in Fe_41_Mn_30_Ga_29_ microwire evolve with increasing magnetic field (from 0.05 to 50 kOe). Specifically, the spontaneous magnetization at T_Mf_ increases from approximately 1.5 to 80.5 emu/g, and the *ΔT*_*hys*_ widens significantly from −5 to 55 K. These results confirm that the low-temperature ferromagnetic martensitic transformation—closely associated with the magnetic shape memory (FSM) effect—can be actively tuned by applying an external magnetic field along the wire axis. As the Mn content is further reduced: In Fe_43_Mn_28_Ga_29_ microwires, the spontaneous magnetization at *T*_*Mf*_ increases from 10 to 83 emu/g, and *ΔT*_*hys*_ increases from −5 to 89 K over the sample field range (0.05 – 50 kOe). In Fe_45_Mn_26_Ga_29_ microwires, the spontaneous magnetization at T_Mf_ increases from 9 to 106 emu/g, while *ΔT*_*hys*_ increases from −6 to 99 K. The negative sign indicates that the magnetization curve obtained during the heating process lies below that of the cooling process. These enhancements in both magnetization and thermal hysteresis with decreasing Mn content highlight that the field-induced FSM behavior can be sensitively controlled by small compositional changes, demonstrating the critical role of Mn concentration in tuning the functional properties of these Heusler-type microwires.^23^

It is important to note that the *M­(T)* curves measured under magnetic fields ranging from 0.05 to 50 kOe exhibit not only distinct *ΔT*_*hys*_ magnitudes but also noticeable differences in curve shape. In contrast, for compositions with *x* = 6, 4, and 2 at. %, the *M*(*T*) curves measured at a low magnetic field (H = 0.05 kOe) display two distinct changes during cooling and heating processes. These distinct features indicate the presence of strong magnetocrystalline anisotropy (MA) in the martensitic phase.^8,9^ According to a previous report,^23^ the MA is relatively large (7.6 × 10^– 5^ J·m^– 3^ at 300 K) compared to other Heusler compounds. The pronounced MA is attributed to the motion of martensitic variant boundaries, highlighting the fact that larger MA could be achieved when the Heusler compounds exhibit both high magnetization and a large c/a ratio value. Our magnetization data clearly show that the magnetization at the onset of the martensitic transformation increases with decreasing Mn content (*x* = 6, 4, and 2 at. %), suggesting that the MA may eventually also increase as *x* decreases. Particularly, the rearrangement of martensite variants is expected to be governed by the volume proportions of Fe and Mn. Higher MA promotes more effective variant selection, thereby enhancing magnetically induced strain and improving the functional characteristics of Fe–Mn–Ga microwires. Interestingly, these anomalies disappear at higher magnetic fields, which is consistent with the expected behavior for martensitic inclusions with magneto-crystalline anisotropy. In such cases, a sufficiently strong magnetic field can overcome the anisotropy energy, effectively aligning magnetic domains and smoothing out the transformation behavior. Based on these observations: (i) the amplitude of the magnetization increases significantly, from 1.5 to 105 emu/g, (ii) the *ΔT*_*hys*_’s width expands from −5 to 99 K, and (iii) the sharpness of the curve inclination (i.e., slope of the transition) increases with applied field, reflecting a more abrupt transformation. The trends collectively indicate that the martensitic and austenite transformations are magnetically driven, and that the energy stored in magnetically induced strain increases with both higher magnetic field strength (H = 0.05–50 kOe) and lower Mn content (*x* = 6–2 at. %).^24^ The results demonstrate that the *ΔT*_*hys*_ associated with the martensitic-to-austenite phase transition is strongly influenced by atomic rearrangements induced by magnetic-field-induced strain, which provides clear evidence of the tunable magneto-structural coupling in off-stoichiometric Fe–Mn–Ga microwires. Figure 3f shows the shift of the transformation temperature (ΔT_hys_) with an applied magnetic field (ΔH), which can be explained on the basis of the Clausius–Clapeyron equation. The equation is expressed as^25,26^

$$\frac{dT\left(H\right)}{dH}=\frac{\Delta M\left(T_{0}\left(H\right),H\right)}{\Delta S(T_{0}\left(H\right),H}$$

where T_0_(H) = (T_Ms_(H) + T_Af_(H))/2 is the equilibrium temperature under a magnetic field, ΔM­(T_0_(H), H) = M^L^(T_0_(H), H) – M^H^(T_0_(H), H) is the difference in magnetization between a low-temperature phase M^L^(T_0_(H), H) and a high-temperature phase M^H^(T_0_(H), H) at a temperature of T_0_(H), under a magnetic field of H, and ΔS­(T_0_(H), H) is the difference in entropy between the two phases at T_0_(H). Typically, the austenite phase in Fe–Mn–Ga exhibits higher magnetization compared with the martensitic phase. Consequently, the application of H stabilizes the austenite phase, resulting in a decrease in the martensitic transformation temperature. This magnetic-field-induced transformation reflects the strong coupling between magnetic and structural degrees of freedom in Fe–Mn–Ga alloys.

In microwires, ΔT_hys_ is further influenced by the internal strain fields and energy barriers arising from the off-stoichiometry and magnetic disorder. Off-stoichiometric deviations introduce lattice distortions and defect structures, which generate heterogeneous internal strain fields acting as pinning centers for the martensitic transformation. These strain fields increase the energy barrier for phase nucleation and propagation, thereby broadening the hysteresis and influencing the sensitivity of ΔT_hys_ to magnetic fields.^27^ Competing ferromagnetic and antiferromagnetic interactions alter the free energy landscape, affecting the magnetic phase stability and entropy change (ΔS). The complex interplay between these interactions results in a distribution of local energy barriers that governs the kinetics of the transformation and the magneto-thermodynamic response, contributing to the observed variations in ΔT_hys_.^28^

Further insight into the magnetic switching behavior and exchange bias characteristics, associated with intrinsic disorder resulting from the off-stoichiometry of Heusler microwires, can be obtained through magnetization-magnetic field (*M*-*H*) hysteresis loop measurements. Figure 4a–c presents the *M-H* loops of glass-coated Fe_47‑x_Mn_24*+x*_Ga_29_ (*x* = 0, 2, 4, 6, 8 at. %) microwires, measured over a field range of −85 kOe to 85 kOe at three different temperatures, 5, 150, and 300 K. At 5 K, all off-stoichiometry microwires exhibit nonsaturating M-H behavior even under a higher applied magnetic field of 85 kOe, confirming the presence of antiferromagnetic state. The loop also exhibits open hysteresis, characteristic of competing ferromagnetic and antiferromagnetic exchange interaction. The coercive field (*H*_*c*_ calculated as |*H*_*c1*_+*H*_*c2*_|/2, where *H*_*c1*_ and *H*_*c2*_ are the right and left intercepts of the loop on the field axis) varied significantly, ranging from 141 and 659 Oe, as shown in Figure 4d. Interestingly, despite the absence of a martensitic transformation for *x* = 8 and 0 at. % compositions, these samples exhibit relatively large coercive fields of 659 and 623 Oe, respectively. In contrast, the compositions with *x* = 6, 4, and 2 at. %, which do undergo magneto-structural transformations, show lower coercive fields that increase slightly with decreasing Mn content, from 141 (*x* = 6) to 412 Oe (*x* = 2 at. %). The remanent magnetization (*M*_*r*_, defined as |*M*_*r1*_+*M*_*r2*_|/2, where *M*_*r1*_ and *M*_*r2*_ are the magnetization values at zero applied field on descending and ascending branches of the loop) also varies with composition. As shown in Figure 4e, *M*_*r*_, increases from approximately 5 to 47 emu/g as Mn content decreases from *x* = 8–0 at. %. For the samples exhibiting martensitic transformations, M_r_ increases more gradually, from 5 to 15.5 emu/g, suggesting a link between structural phase change and soft magnetic behavior. These observations support the conclusion that microwires undergoing martensitic transformations exhibit softer magnetic behavior characterized by low coercivity and moderate remanence. In contrast, magnetically harder microwire, which do not exhibit martensitic transformation (e.g., *x* = 0 and 8 at. %), display stronger coercivity a higher remanence—implying that the realization of a martensitic transition is more favorable in magnetically softer materials.

**Figure 4:**
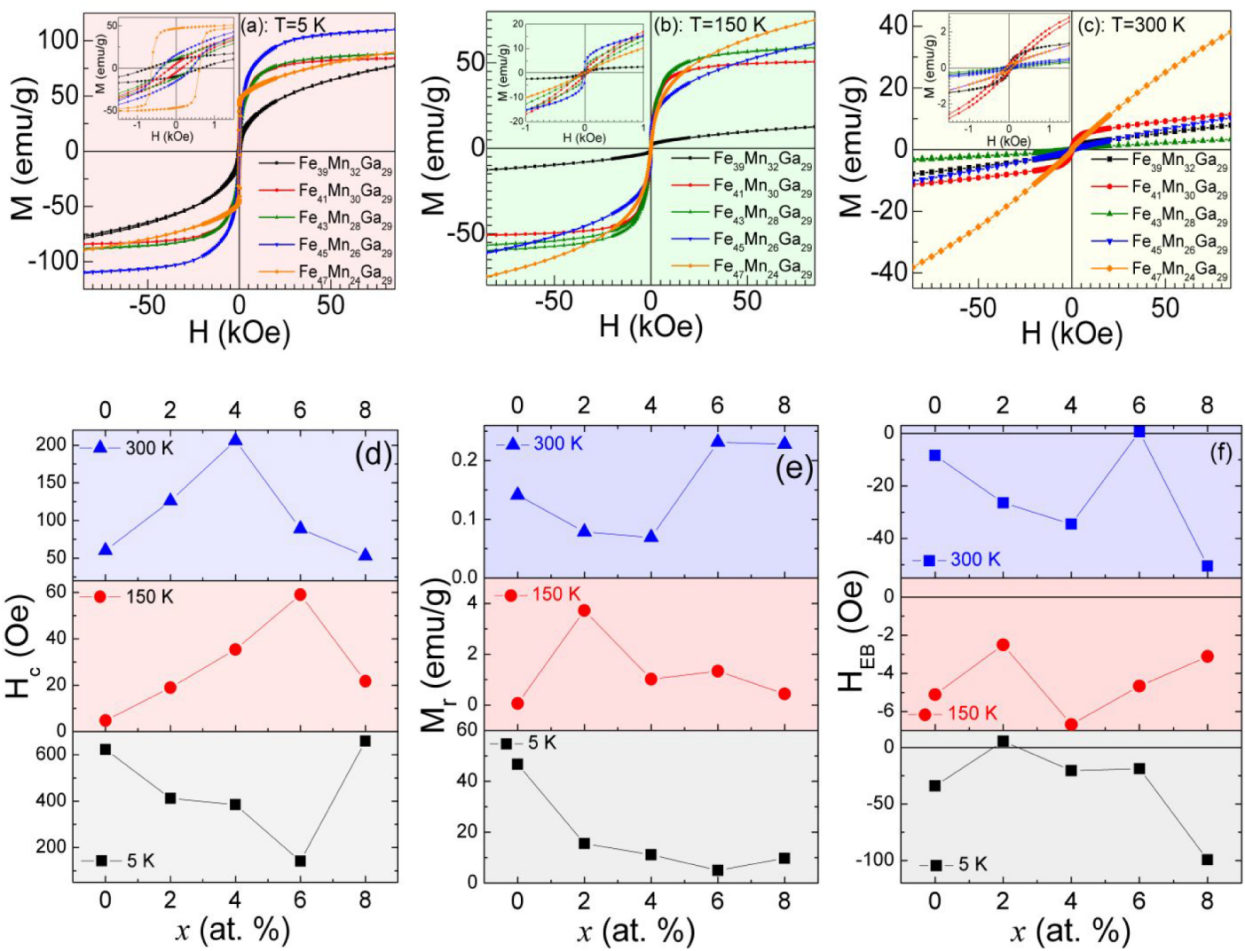
Comparison of magnetic hysteresis loops of Fe_1–*x*_Mn_*x*_Ga_29_ glass-coated microwires measured at (a) *T* = 5 K (ferrimagnetic state), (b) 150 K (ferromagnetic state), and (c) 300 K (weak ferromagnetic/paramagnetic state). (df) *x* dependence of coercive field *(H*_*C*_), remanence magnetization *(M*_*r*_), and exchange bias effects *(H*_*EB*_).

From Figure 4b, it is evident that both the left and right *H*_*c*_ begin to approach zero as the temperature increases to 150 K, corresponding to the coupled magneto-structural transformation regime. The average *H*_*c*_ value decreases from 59 to 4.75 Oe as the Mn content decreases from *x* = 8 to 0 at. %. This variation (∼54 Oe) is over 10 times smaller than the coercivity measured at 5 K, highlighting the softening of magnetic properties near the transformation temperature. At 150 K, the remanent magnetization *M*_*r*_ also varies with the composition. As Mn content decreases from *x* = 8–2 at. %, *M*_*r*_ increases from 0.4 to 3.72 emu/g, followed by a sharp decrease from 3.7 to 0.06 emu/g as *x* decreases further to 0 at. %. The magnetization at 85 kOe increases from 12.4 to 75.3 emu/g as *x* decreases from 8 and 0 at. %, with compositions *x* = 6 and 4 at. % reaching saturation values of 50.5 and 58.9 emu/g, respectively. At 300 K, as shown in Figure 4c, both the left and right *H*_*c*_ values continue to shift toward zero. However, the *H*_*c*_ values vary between 53 and 206 Oe depending on composition. Notably, the microwire that exhibits martensitic transformation (*x* = 2–6 at. %) possesses relatively high *Hc* values (89–206 Oe), which are significantly larger than those observed at 150 K. At this temperature, *M*_*r*_ shows a modest variation, ranging from 0.07 to 0.23 emu/g as *x* changes from 8 to 0 at. %. While samples with *x* = 8–4 at % exhibit spontaneous magnetization under an external magnetic field, compositions *x* = 0–2 at. % show an almost complete absence of ferromagnetic ordering. These temperature- and composition-dependent M­(H) loops at 150 K indicate the narrow hysteresis loops and low coercivity, and this magnetic softness in Fe–Mn–Ga alloy can be closely associated with (i) domain wall mobility—domain wall mobility plays a dominant role in governing the magnetization process. The reduced magneto-crystalline anisotropy in certain martensitic variants facilitates easier domain wall motion under an applied magnetic field, thereby contributing to soft magnetic behavior.^29–31^ The presence of structural defects and residual stresses introduced during the wire-drawing process can significantly affect the alloy’s magnetic properties. In particular, such imperfections may hinder domain wall motion and alter the magnetic hysteresis behavior. (ii) Pinning centers—such as lattice defects, chemical disorder, or internal stresses introduced during the phase transformation—can locally impede domain wall motion. While excessive pinning would increase coercivity, the relatively low coercivity observed here suggests that the pinning landscape is weak and sparsely distributed, allowing for reversible domain wall motion with minimal energy loss. (iii) Twin boundaries, intrinsic to the martensitic microstructure of Fe–Mn–Ga, have a dual effect. First, they can act as a barrier to domain wall propagation, slightly resisting magnetic reversal. On the other hand, they can serve as easy pathways for domain wall nucleation and motion, depending on their crystallographic orientation. The balance between these opposing effects appears to favor enhanced wall mobility, thus maintaining the magnetic softness.^32^

In Figure 4e, the exchange bias field *H*_*EB*_, calculated as *H*_*EB*_ = (*H*_*c1*_-*H*_*c2*_)/2, is plotted against Mn content (*x* = 8–0 at. %) for three temperatures: 5, 150, and 300 K. The *M­(H)* loop shows both positive and negative *H*_*EB*_ shifts, typically along the field axis, representing positive and negative H_EB_ effects, respectively. At 300 K, negative *H*_*EB*_ values range from −8.4 to −50 Oe as x decreases from *x* = 8 to 0 at. %. Interestingly, a positive *H*_*EB*_ of +0.6 Oe is observed for *x* = 2 at. %, suggesting subtle shifts in interfacial magnetic interactions. At *T* = 150 K, which is near the martensitic transformation temperature (*T* < T_Ms_ or T_Af_), the negative H_EB_ value varies moderately between −2.5 to −6.7 Oe across the composition range. At *T* = 5 K (<T_N_), the H_EB_ decreases more significantly, from −99 → −18 Oe with decreasing Mn content (*x* = 8 → 0 at %). A positive *H*_*EB*_ of +5.6 Oe is again noticed for *x* = 2 at. %. The sign reversal at this critical composition can be explained by the following factors: (i) the positive H_EB_ suggests a change in the dominant exchange mechanism at the interface, possibly caused by structural modifications that alter spin alignment.^33^ (ii) Mn substitution in place of Fe or defects may lead to a reorganization of pinned interfacial spins, favoring a new domain structure that produces a positive rather than a negative bias.^34^ (iii) Although the first hysteresis loop shows a positive H_EB_, subsequent loops display a negative H_EB_. This indicates that the positive H_EB_ is likely metastable, resulting from a nonequilibrium interfacial state that relaxes with repeated cycling.^35^ The observation of large negative *H*_*EB*_ and nonsaturating magnetization up to 85 kOe confirm the presence of dominant Mn–Mn and Mn–Fe intersublattice antiferromagnetic exchange interactions, further supported by *ac* susceptibility measurements. The origin of positive and negative zero-field-cooled *H*_*EB*_ effects can be attributed to interfacial exchange interactions between ferromagnetic (*FM*) and antiferromagnetic (*AFM*) regions, driven by exchange anisotropy.^36,37^ The gradual decrease in *H*_*EB*_ is explained by the growth of the Fe–Mn AFM phase due to spin rearrangements, which in turn reduced exchange pinning efficiency at the FM/AFM interface. Notably, very small negative *H*_*EB*_ values (>-7 Oe) observed below the martensitic transformation temperature in ferromagnetic phases may be due to gradual atomic position changes and weakening of Mn–Mn AFM interactions, likely resulting from interface disorder and reduced domain wall pinning in the FM/AFM regions.

To gain deeper insight into the magnetic interactions governing the complex phase transitions in the system, dynamic *ac* magnetic susceptibility measurements were performed on glass-coated Fe_47‑*x*_Mn_24+*x*_Ga_29_ microwires in the absence of a *dc* magnetic field. Figure 5a-b shows the zero-field-cooled temperature dependence of the in-plane real part (χ’) and out-of-plane imaginary part (χ″) of the *ac* susceptibility for compositions *x* = 8, 6, 4, 2, and 0 at. %. The measurements were conducted using ac field of *h*_ac_ = 10 Oe and excitation frequency *f* = 1 kHz applied along the longitudinal axis of the microwires (growth direction). Both the χ’(*T*) and χ”(*T*) spectra exhibit similar temperature-dependent trends, with a sharp rise upon cooling between 220 and 120 K. This behavior is indicative of a magnetic phase transition from the high-temperature paramagnetic (PM) state to a low-temperature ferromagnetic (FM) state, where long-range spin alignment becomes dominant. For the *x* = 8 and 0 at. % samples, the Curie temperatures (*T*_c_) were estimated from the inflection points in the χ’(*T*) and χ”(*T*) curves to be approximately 175 and 142 K, respectively. Moreover, for compositions *x* = 6, 4, and 2 at. %, both χ’(*T*) and χ”(*T*) spectral features shift gradually toward low temperatures as Mn content decreases. This trend is indicative of a composition-dependent martensitic start temperature, T_Ms_, which varies from 184 to 154 K, as shown in Figure 5c. The *T*_c_ (for x = 8 and 0 at. %) and *T*_Ms_ (for *x* = 2, 4, and 6 at. %) values correlate well with the results obtained from *M*(*T*) measurements, thereby confirming the reliability of the magnetic phase characterization. The observed variation in transition temperatures with Mn content can be attributed to the partial substitution of Mn *s* and *d* orbitals with Fe *s* and *d* orbitals, which enhance the exchange interactions and stabilize long-range ferromagnetic ordering. The progressive reduction in *T*_c_ and T_Ms_ with increasing Fe content suggests a delicate balance between ferromagnetic exchange and structural staining, likely modulated by the local atomic configuration and electronic hybridization within the off-stoichiometric Heusler phase. The χ″(*T*) reveals critical insights into the magnetic energy dissipation mechanisms within the material. As shown in Figure 5b, χ″ exhibits pronounced peaks near 150 K, indicating energy loss associated with magnetic relaxation processes. This peak corresponds to an increase in magnetic damping, which may arise from domain wall motion impeded by pining centers, spin reorientation phenomena, or phase coexistence effects. The sharp decline in χ” above 150 K suggests a magnetic phase transition, likely from a ferromagnetic to paramagnetic state where magnetic energy dissipation is substantially reduced. At temperatures below 20 K and above 200 K, the low values of χ” indicate minimal magnetic losses, consistent with stable magnetic ordering and reduced dynamic magnetic processes. These observations collectively highlight the intricate interplay between magnetic transitions and energy dissipation mechanisms.^38,39^

**Figure 5:**
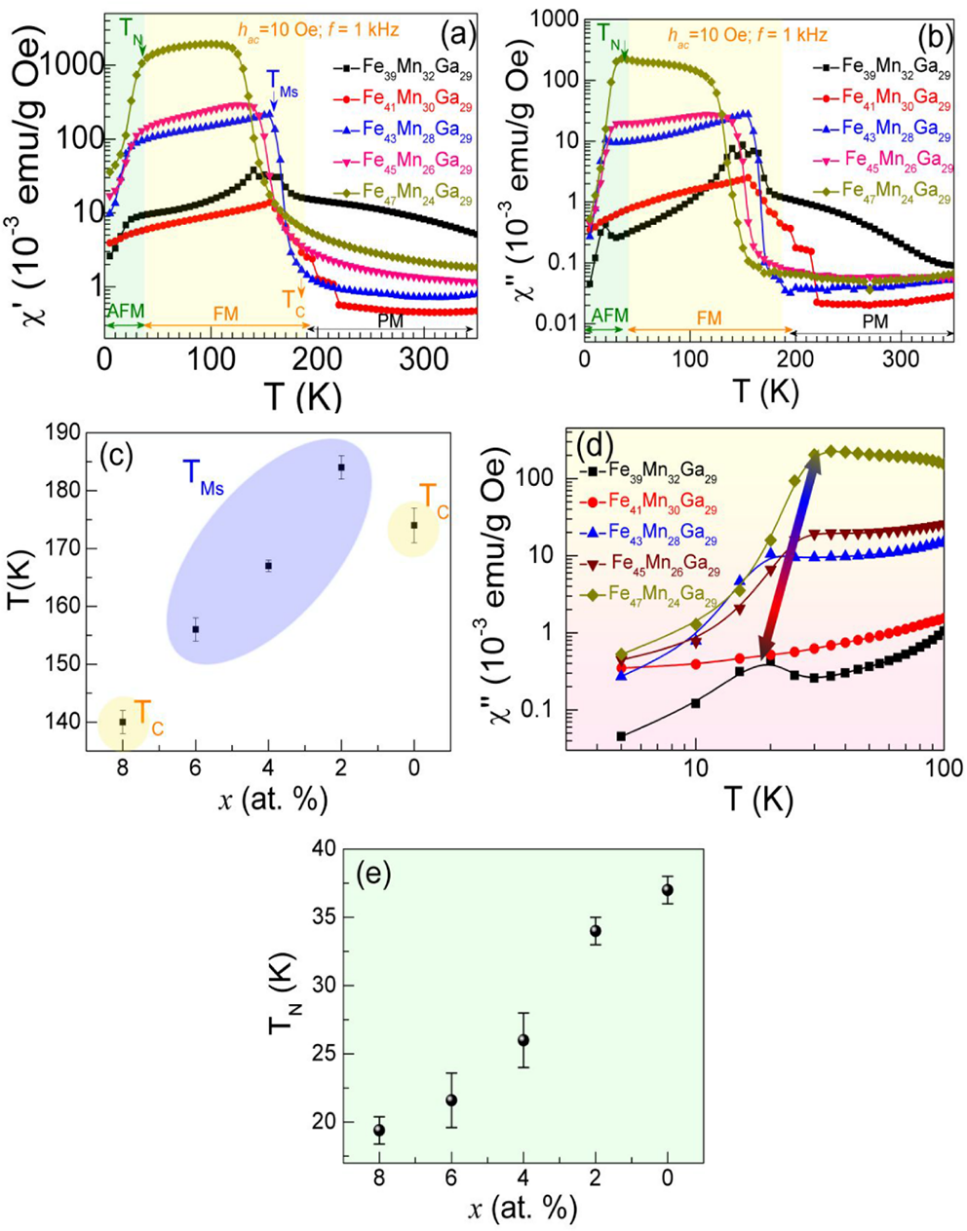
(a, b) Temperature dependence of the real χ’ (in-plane) and imaginary χ” (out-of-plane) components of the *ac* magnetic susceptibility of Fe_47‑x_Mn_24+x_Ga_29_ microwires measured with a frequency *f* = 1 kHz and an amplitude of *ac* field *h*_ac_ = 10 Oe. (c) *T*_*c*_ and *T*_*Ms*_ values plotted against the atomic percentage (*x* at. %). (d) Temperature dependence of χ” showing the shift of *T*_*N*_ with decreasing *x* at. %. (e) *T*_***N***_ as a function of *x* (at. %).

It is important to note that the high-temperature ferromagnetic transitions are commonly observed in off-stoichiometry Heusler alloys, while low-temperature antiferromagnetic (AFM) transitions have not been reported previously to the best of our knowledge.^15^ The representative χ’(*T*) and χ”(*T*) curves display a gradual decrease from 130 to 40 K, followed by a sharper decrease below 40 K, with a distinct inflection point emerging in the low-temperature region. The inflection is indicative of a secondary magnetic phase transition, likely corresponding to the Neel temperature, T_N_, marking the onset of antiferromagnetic (AFM) ordering.^40,41^ This form of transition has been largely emerging due to competing ferromagnetic and AFM exchange interaction between neighboring Fe and Mn atoms, especially in compositions with Mn concentrations exceeding 25%.^41^ As shown in Figure 5d, the low-temperature features of χ″(*T*) curves exhibit significant variations in line shape and peak position across different compositions. These features reveal several key observations: (i) Below *T*_*N*_, χ”(T) shows a monotonic decrease, accompanied by a narrowing of the AFM-related peak, suggesting that the applied *ac* magnetic field (*h*_ac_ = 10 Oe) is aligned parallel to the (220) crystallographic plane;^41^ (ii) the *T*_*N*_ peak shifted toward higher temperatures as Mn content increases, indicating a thermally driven stabilization of the AFM phase; and (iii) the magnitude of the AFM peak [χ”(T)] increases by more than 2 orders of magnitude as the Mn content increases from *x* = 0 to 8 at. %, demonstrating composition-dependent enhancement of AFM interactions. Specifically, the *T*_*N*_ shifts from 19 to 37 K with increasing Mn content marked by the green arrows in Figure 5d. These findings strongly suggest that the AFM transition is governed by the strength of exchange interactions arising from the relative volume fractions of Fe and Mn atoms. The composition-dependent *T*_*N*_ values are summarized in Figure 5e. It is also worth noting that increasing Mn content (and thereby reducing Fe content) causes a thermal downshift of the AFM phase, likely due to exchange bias effects arising from pinned FM spins at the FM/AFM interface in the presence of crystalline anisotropy.^40^ These interactions drive local rearrangements of atomic moments, including Mn antisite disorder, and affect the thermodynamic stability of the ferromagnetic shape memory (FSM) phase. Furthermore, *M–T* measurements confirm the reversibility of AFM transitions below 37 K, with only minor deviations between the cooling and heating curves. The observed behavior aligns with previous findings and supports a complex magnetic phase diagram in Fe–Mn–Ga-based Heusler alloys, where a low-temperature AFM phase coexists with or is embedded in a dominant FM background. In conclusion, the emergence of this low-temperature AFM transition is best explained by dynamic AFM clustering, which plays a central role in the FM/AFM exchange coupling and underpins the exchange bias effect observed in these microwires. The fact that this AFM behavior is visible in both temperature-dependent magnetization and *ac* susceptibility measurements—even under very low magnetic fields—indicates that it is a thermodynamically second-order transition, in which the thermal average of magnetic moments evolves continuously upon cooling through *T*_*N*_.

In summary, we have investigated the thermally and magnetically activated FMS response of glass-coated Fe_47‑*x*_Mn_24+*x*_Ga_29_ (*x* = 8–0 at. %) microwires over an extended temperature range encompassing both martensitic and austenite phases. Magnetization measurements during cooling and heating cycles reveal thermal hysteresis, from which we evaluated the variation in its magnitude and shape as a function of Mn and Fe content and applied magnetic fields. Notably, the Fe_39_Mn_32_Ga_29_ and Fe_47_Mn_24_Ga_29_ microwires exhibited no thermal hysteresis even under an applied magnetic field of 50 kOe, indicating that FMS is completely suppressed—probably due to the mechanically soft and brittle nature of these compositions. For the Fe_45_Mn_26_Ga_29_ microwire, a large thermal hysteresis width *ΔT*_*Hys*_ of up to 98 K was observed. The temperature dependence of both in-plane and out-of-plane *ac* susceptibility showed monotonic decreases below a critical point, suggesting the emergence of an AFM. This is attributed to unequal filling of 3*d* orbitals in Fe and Mn ions. The *T*_*N*_ was found to shift from 22 to 41 K, explained by the exchange interaction between the neighboring Mn–Mn and Fe–Mn ions. The observed correlation between Mn substitution and magnetic memory response demonstrates that precise compositional tuning can control magneto-structural characteristics. These findings highlight the promising potential of low-cost Fe–Mn–Ga microwires for use in magnetic actuators and sensing technologies

## ∇

Instituto de Física Gleb Wataghin, Universidade Estadual de Campinas, 13083-859, Campinas, São Paulo, Brazil
